# Supplementary material for: Evaluation of a novel, rapid antigen detection test for the diagnosis of SARS-CoV-2
Source: PLoS One. 2021 Nov 29;16(11):e0259527. doi: 10.1371/journal.pone.0259527 (PMC8629250; doi:10.1371/journal.pone.0259527)
Supplement: S1 File — (DOCX) [file pone.0259527.s001.docx]

**SUPPLEMENT**

| female | | Rt-PCR | | Total |
| --- | --- | --- | --- | --- |
|  |  | negative | positive |  |
| AG-rt | negative | 189 | 21 | 210 |
|  | positive | 2 | 90 | 92 |
| Total | | 191 | 111 | 302 |

| **Statistic** | **Value** | **95% CI** |
| --- | --- | --- |
| **Sensitivity** | 81.08% | 72.55% to 87.89% |
| **Specificity** | 98.95% | 96.27% to 99.87% |
| **Positive Likelihood Ratio** | 77.43 | 19.45 to 308.27 |
| **Negative Likelihood Ratio** | 0.19 | 0.13 to 0.28 |
| **Disease prevalence (*)** | 36.75% | 31.31% to 42.47% |
| **Positive Predictive Value (*)** | 97.83% | 91.87% to 99.44% |
| **Negative Predictive Value (*)** | 90.00% | 85.96% to 92.97% |
| **Accuracy (*)** | 92.38% | 88.79% to 95.11% |

| male | | Rt-PCR | | Total |
| --- | --- | --- | --- | --- |
|  |  | negative | positive |  |
| AG-rt | negative | 136 | 21 | 157 |
|  | positive | 1 | 81 | 82 |
| Total | | 137 | 102 | 239 |

| **Statistic** | **Value** | **95% CI** |
| --- | --- | --- |
| **Sensitivity** | 79.41% | 70.27% to 86.78% |
| **Specificity** | 99.27% | 96.00% to 99.98% |
| **Positive Likelihood Ratio** | 108.79 | 15.40 to 768.77 |
| **Negative Likelihood Ratio** | 0.21 | 0.14 to 0.30 |
| **Disease prevalence (*)** | 42.68% | 36.32% to 49.22% |
| **Positive Predictive Value (*)** | 98.78% | 91.98% to 99.83% |
| **Negative Predictive Value (*)** | 86.62% | 81.56% to 90.46% |
| **Accuracy (*)** | 90.79% | 86.40% to 94.14% |

| young (18-45y) | | Rt-PCR | | Total |
| --- | --- | --- | --- | --- |
|  |  | negative | positive |  |
| AG-rt | negative | 166 | 12 | 178 |
|  | positive | 0 | 73 | 73 |
| Total | | 137 | 166 | 85 |

| **Statistic** | **Value** | **95% CI** |
| --- | --- | --- |
| **Sensitivity** | 85.88% | 76.64% to 92.49% |
| **Specificity** | 100.00% | 97.80% to 100.00% |
| **Positive Likelihood Ratio** |  |  |
| **Negative Likelihood Ratio** | 0.14 | 0.08 to 0.24 |
| **Disease prevalence (*)** | 33.86% | 28.03% to 40.08% |
| **Positive Predictive Value (*)** | 100.00% |  |
| **Negative Predictive Value (*)** | 93.26% | 89.12% to 95.90% |
| **Accuracy (*)** | 95.22% | 91.80% to 97.51% |

| middle (46-65y) | | Rt-PCR | | Total |
| --- | --- | --- | --- | --- |
|  |  | negative | positive |  |
| AG-rt | negative | 87 | 20 | 107 |
|  | positive | 2 | 55 | 57 |
| Total | | 89 | 75 | 164 |

| **Statistic** | **Value** | **95% CI** |
| --- | --- | --- |
| **Sensitivity** | 73.33% | 61.86% to 82.89% |
| **Specificity** | 97.75% | 92.12% to 99.73% |
| **Positive Likelihood Ratio** | 32.63 | 8.23 to 129.33 |
| **Negative Likelihood Ratio** | 0.27 | 0.19 to 0.40 |
| **Disease prevalence (*)** | 45.73% | 37.94% to 53.68% |
| **Positive Predictive Value (*)** | 96.49% | 87.40% to 99.09% |
| **Negative Predictive Value (*)** | 81.31% | 74.90% to 86.38% |
| **Accuracy (*)** | 86.59% | 80.40% to 91.40% |

| old (66y plus) | | Rt-PCR | | Total |
| --- | --- | --- | --- | --- |
|  |  | negative | positive |  |
| AG-rt | negative | 72 | 10 | 82 |
|  | positive | 1 | 43 | 44 |
| Total | | 73 | 53 | 126 |

| **Statistic** | **Value** | **95% CI** |
| --- | --- | --- |
| **Sensitivity** | 81.13% | 68.03% to 90.56% |
| **Specificity** | 98.63% | 92.60% to 99.97% |
| **Positive Likelihood Ratio** | 59.23 | 8.42 to 416.64 |
| **Negative Likelihood Ratio** | 0.19 | 0.11 to 0.33 |
| **Disease prevalence (*)** | 42.06% | 33.33% to 51.18% |
| **Positive Predictive Value (*)** | 97.73% | 85.94% to 99.67% |
| **Negative Predictive Value (*)** | 87.80% | 80.46% to 92.64% |
| **Accuracy (*)** | 91.27% | 84.92% to 95.56% |

| fever | | Rt-PCR | | Total |
| --- | --- | --- | --- | --- |
|  |  | negative | positive |  |
| AG-rt | negative | 122 | 29 | 151 |
|  | positive | 3 | 109 | 112 |
| Total | | 125 | 138 | 263 |

| **Statistic** | **Value** | **95% CI** |
| --- | --- | --- |
| **Sensitivity** | 78.99% | 71.23% to 85.45% |
| **Specificity** | 97.60% | 93.15% to 99.50% |
| **Positive Likelihood Ratio** | 32.91 | 10.72 to 100.99 |
| **Negative Likelihood Ratio** | 0.22 | 0.16 to 0.30 |
| **Disease prevalence (*)** | 52.47% | 46.25% to 58.64% |
| **Positive Predictive Value (*)** | 97.32% | 92.21% to 99.11% |
| **Negative Predictive Value (*)** | 80.79% | 75.25% to 85.34% |
| **Accuracy (*)** | 87.83% | 83.26% to 91.53% |

| cough | | Rt-PCR | | Total |
| --- | --- | --- | --- | --- |
|  |  | negative | positive |  |
| AG-rt | negative | 137 | 27 | 164 |
|  | positive | 3 | 97 | 100 |
| Total | | 140 | 124 | 264 |

| **Statistic** | **Value** | **95% CI** |
| --- | --- | --- |
| **Sensitivity** | 78.23% | 69.92% to 85.13% |
| **Specificity** | 97.86% | 93.87% to 99.56% |
| **Positive Likelihood Ratio** | 36.51 | 11.87 to 112.25 |
| **Negative Likelihood Ratio** | 0.22 | 0.16 to 0.31 |
| **Disease prevalence (*)** | 46.97% | 40.82% to 53.18% |
| **Positive Predictive Value (*)** | 97.00% | 91.32% to 99.00% |
| **Negative Predictive Value (*)** | 83.54% | 78.41% to 87.64% |
| **Accuracy (*)** | 88.64% | 84.18% to 92.20% |

| dyspnoea | | Rt-PCR | | Total |
| --- | --- | --- | --- | --- |
|  |  | negative | positive |  |
| AG-rt | negative | 82 | 12 | 94 |
|  | positive | 1 | 45 | 46 |
| Total | | 83 | 57 | 140 |

| **Statistic** | **Value** | **95% CI** |
| --- | --- | --- |
| **Sensitivity** | 78.95% | 66.11% to 88.62% |
| **Specificity** | 98.80% | 93.47% to 99.97% |
| **Positive Likelihood Ratio** | 65.53 | 9.30 to 461.84 |
| **Negative Likelihood Ratio** | 0.21 | 0.13 to 0.35 |
| **Disease prevalence (*)** | 40.71% | 32.50% to 49.33% |
| **Positive Predictive Value (*)** | 97.83% | 86.46% to 99.69% |
| **Negative Predictive Value (*)** | 87.23% | 80.51% to 91.87% |
| **Accuracy (*)** | 90.71% | 84.64% to 94.96% |

| sore throat | | Rt-PCR | | Total |
| --- | --- | --- | --- | --- |
|  |  | negative | positive |  |
| AG-rt | negative | 143 | 12 | 155 |
|  | positive | 0 | 51 | 51 |
| Total | | 143 | 63 | 206 |

| **Statistic** | **Value** | **95% CI** |
| --- | --- | --- |
| **Sensitivity** | 80.95% | 69.09% to 89.75% |
| **Specificity** | 100.00% | 97.45% to 100.00% |
| **Positive Likelihood Ratio** |  |  |
| **Negative Likelihood Ratio** | 0.19 | 0.11 to 0.32 |
| **Disease prevalence (*)** | 30.58% | 24.37% to 37.36% |
| **Positive Predictive Value (*)** | 100.00% |  |
| **Negative Predictive Value (*)** | 92.26% | 87.75% to 95.20% |
| **Accuracy (*)** | 94.17% | 90.05% to 96.95% |
