# Supplementary material for: Evaluation of a novel, rapid antigen detection test for the diagnosis of SARS-CoV-2
Source: PLoS One. 2021 Nov 29;16(11):e0259527. doi: 10.1371/journal.pone.0259527 (PMC8629250; doi:10.1371/journal.pone.0259527)
Supplement: Attachment — Submitted filename: Response to Reviewers BLv20210707.docx [file pone.0259527.s002.docx]

Dear Editor-in-Chief,

Dear Reviewers,

Thanks for your constructive suggestions. We have changed and answered each point (see below) to the best of our knowledge.

Kind regards,

Rainer Thell

**Journal Requirements:**

When submitting your revision, we need you to address these additional requirements.

1. Please ensure that your manuscript meets PLOS ONE's style requirements, including those for file naming. The PLOS ONE style templates can be found at [**https://journals.plos.org/plosone/s/file?id=wjVg/PLOSOne_formatting_sample_main_body.pdf**](https://journals.plos.org/plosone/s/file?id=wjVg/PLOSOne_formatting_sample_main_body.pdf) and [https://journals.plos.org/plosone/s/file?id=ba62/PLOSOne_formatting_sample_title_authors_affiliations.pdf [2](https://journals.plos.org/plosone/s/file?id=ba62/PLOSOne_formatting_sample_title_authors_affiliations.pdf%20%5b2)]

2. Please review your reference list to ensure that it is complete and correct. If you have cited papers that have been retracted, please include the rationale for doing so in the manuscript text, or remove these references and replace them with relevant current references. Any changes to the reference list should be mentioned in the rebuttal letter that accompanies your revised manuscript. If you need to cite a retracted article, indicate the article's retracted status in the References list and also include a citation and full reference for the retraction notice.

AW: We are unaware any of the cited paper to be retracted.

3. Please include the full name of the ethics committee(s) that approved your study in the manuscript Methods.

AW: We included the full names in the manuscript.

4. PLOS requires an ORCID iD for the corresponding author in Editorial Manager on papers submitted after December 6th, 2016. Please ensure that you have an ORCID iD and that it is validated in Editorial Manager. To do this, go to 'Update my Information' (in the upper left-hand corner of the main menu), and click on the Fetch/Validate link next to the ORCID field. This will take you to the ORCID site and allow you to create a new iD or authenticate a pre-existing iD in Editorial Manager. Please see the following video for instructions on linking an ORCID iD to your Editorial Manager account: https://www.youtube.com/watch?v=_xcclfuvtxQ

AW: The corresponding author’s ORCID has been included/updated.

5. Please upload a copy of Supplement Table S1 which you refer to in your text on page 9.

AW: We uploaded the Supplement. We also deleted ‘Table S1’ and left only ‘Supplement’ in the manuscript, since there were more than one table presenting mentioned data!

6. Thank you for stating the following in the Funding Section of your manuscript: "Roche diagnostics provided the SARS-CoV-2 Rapid Antigen Test (Roche Diagnostics). All authors declare that they have no conflict of interest. Our group received funding for this research project from Roche Diagnostics."

We note that you have provided funding information that is not currently declared in your Funding Statement. However, funding information should not appear in the Acknowledgments section or other areas of your manuscript. We will only publish funding information present in the Funding Statement section of the online submission form.

Please remove any funding-related text from the manuscript and let us know how you would like to update your Funding Statement. Currently, your Funding Statement reads as follows: "no"

Additionally, because some of your funding information pertains to [commercial funding//patents], we ask you to provide an updated Competing Interests statement, declaring all sources of commercial funding.

In your Competing Interests statement, please confirm that your commercial funding does not alter your adherence to PLOS ONE Editorial policies and criteria by including the following statement: "This does not alter our adherence to PLOS ONE policies on sharing data and materials." as detailed online in our guide for authors http://journals.plos.org/plosone/s/competing-interests [2].  If this statement is not true and your adherence to PLOS policies on sharing data and materials is altered, please explain how.

Please include the updated Competing Interests Statement and Funding Statement in your cover letter. We will change the online submission form on your behalf.

Please know it is PLOS ONE policy for corresponding authors to declare, on behalf of all authors, all potential competing interests for the purposes of transparency. PLOS defines a competing interest as anything that interferes with, or could reasonably be perceived as
interfering with, the full and objective presentation, peer review, editorial decision-making, or publication of research or non-research articles submitted to one of the journals. Competing interests can be financial or non-financial, professional, or personal. Competing interests can arise in relationship to an organization or another person. Please follow this link to our website for more details on competing interests: [http://journals.plos.org/plosone/s/competing-interests [2](http://journals.plos.org/plosone/s/competing-interests%20%5b2)]

AW: Please include our updates regarding Competing Interests Statement and Funding Statement. Thanks.

**Funding section**

Roche diagnostics provided the SARS-CoV-2 Rapid Antigen Test (Roche Diagnostics) and limited funding for this research project.

**Competing Interests section**

The authors declare no conflict of interest since Roche diagnostics had no influence on study design, data collection, interpretation of the results, or the final manuscript.

Reviewers' comments:

Reviewer's Responses to Questions
COMMENTS TO THE AUTHOR

1. Is the manuscript technically sound, and do the data support the conclusions?

The manuscript must describe a technically sound piece of scientific research with data that supports the conclusions. Experiments must have been conducted rigorously, with appropriate controls, replication, and sample sizes. The conclusions must be drawn appropriately based on the data presented.
Reviewer #1: Yes
Reviewer #2: Partly
-------------------------

2. Has the statistical analysis been performed appropriately and rigorously?
Reviewer #1: I Don't Know
Reviewer #2: N/A
-------------------------

3. Have the authors made all data underlying the findings in their manuscript fully available?

The PLOS Data policy [3] requires authors to make all data underlying the findings described in their manuscript fully available without restriction, with rare exception (please refer to the Data Availability Statement in the manuscript PDF file). The data should be provided as part of the manuscript or its supporting information, or deposited to a public repository. For example, in addition to summary statistics, the data points behind means, medians and variance measures should be available. If there are restrictions on publicly sharing data--e.g. participant privacy or use of data from a third party--those must be specified.
Reviewer #1: Yes
Reviewer #2: No
-------------------------

4. Is the manuscript presented in an intelligible fashion and written in standard English?
PLOS ONE does not copyedit accepted manuscripts, so the language in submitted articles must be clear, correct, and unambiguous. Any typographical or grammatical errors should be corrected at revision, so please note any specific errors here.
Reviewer #1: Yes
Reviewer #2: Yes
-------------------------

5. Review Comments to the Author
Please use the space provided to explain your answers to the questions above. You may also include additional comments for the author, including concerns about dual publication, research ethics, or publication ethics. (Please upload your review as an attachment if it exceeds 20,000 characters)

Reviewer #1:

This is part of an important body of literature, and should be published by PLOS. However, it should be noted that (like essentially every publication in this field of this type) this paper does not strongly meet the public health need, which boils down to one question: Should we use this test?

The conclusions are in the Results section (which is fine), but the statements in the Conclusion section that the two tests are "comparable" is vacant (obviously, a test that has a PPV of zero can be "compared" to a test with a PPV of 100). Likewise, the statement that "the highest sensitivity was detected with a high viral load" is, of course, unexceptional. The key point of this paper, which is VERY important and MUST be published, likely to be highlighted, is buried in the discussion: "Our results differ from the numbers claimed by the manufacturer, who reported a sensitivity of [line 251] 96.5% and a specificity of 99.7%". We would prefer something more direct, like: "The results reported here suggest poorer performance than claimed by the manufacturer".

AW: We adopted the conclusion as recommended.

The public health questions begin with the recognition that infected persons who might enter a public space have a range of viral loads in their "expectorant", the material that they might broadcast while speaking, coughing, sneezing, exhaling. The risk that they present to the public space is (likely) an increasing function of that viral load. That risk may have a cutoff, that is, a viral load that is high enough to remain detectable by a specific test, but sufficiently low that expectorant does not present a risk. The question that everyone asks about a test being discussed is: Does the test meet the cutoff? Or is the sensitivity of the test so poor that it leaves undetected a fraction of the persons who present a risk.

Of course, COVID-19 is especially problematic in its asymptomatic carriers. This paper, of course, looks at only symptomatic people. The manufacturer perhaps looked at even MORE symptomatic people. Thus, the "statistical analysis" beloved by biostatisticians (and PLOS reviewers/editors) is overwhelmed by a factor not captured, or captur-able, by any statistical analysis. A systematic sampling bias.

Here, this review runs counter to the "culture" in this field. That culture looks for the "statistical analysis". A paper is accepted in the field if it applies correctly a few "tests", reports chi squares, and so on, all beloved by statisticians. As a result we get lots of papers that would get an A grade in a statistics course, but provide little guidance to PHS officials.

Now, I am recommending publication for two reasons:
1. What we PHS people want, we cannot get. Regulatory agencies, IRBs, and the entire "business as usual" government-medical bureaucracy of "experts" place obstacles to the needed studies. Specifically, such studies need to include asymptomatic people selected randomly and by surprise. These are people who are NOT enrolled in studies where filling out paperwork is taken to be the equivalent of "ethics". However, there is no reason to reject this paper and its important conclusion because "experts" are preventing (and have done so for more than a year now) the studies that are needed to manage this pandemic.

2. We do not have an understanding of the function that relates forward transmission risk to viral load. Thus, we do not know the cutoff. This means that even if the "experts" were to allow a truly random sample unfiltered by an IRB-style enrollment program, we could not compare the cutoff (if one exists) with the sensitivity of the test.

Reviewer #2:

The study is evidently, a precise and concise research. It was fascinating as the authors proposed a fast and feasible approach (SARS-CoV-2 Rapid Antigen Test) the SARS-CoV-2 infection can be detected, compared to rt-PCR. The methods that were adopted for the study, including the omission cases where certain criteria were not met by any study participant, was apt and impressive.

However, I have minor concerns that I would love the authors to address for clarifications.

The age group was between 18-90, as the selected population cohort for the study, Is there a possibility this detection method could work for those outside that age group especially children and teenagers. Kindly, give your reasons and if there are future research that will be conducted on that.

AW: The reason for having studied patients just above 18 years of age is that patients were recruited in adult emergency departments (296 of a total 541 patients), were no children are seen.

For the statistical analysis, I would advise the authors to provide a convincing graphical displays in colours to easily different different scenario. This also applies to the flow chart provided by the authors.

AW: Figures are in colours (e.g. see Fig.). Maybe it was not visible as pdf?

Lastly, I observed few grammatical errors and punctutaions.

Asides these, i think it is a good and a novel approach that the authors have adopted.
-------------------------

6. PLOS authors have the option to publish the peer review history of their article (what does this mean? [4]). If published, this will include your full peer review and any attached files.

If you choose "no", your identity will remain anonymous but your review may still be made public.

DO YOU WANT YOUR IDENTITY TO BE PUBLIC FOR THIS PEER REVIEW?

For information about this choice, including consent withdrawal, please see our Privacy Policy [5].

Reviewer #1: No
Reviewer #2: YES: Onyeka S. Chukwudozie

While revising your submission, please upload your figure files to the Preflight Analysis and Conversion Engine (PACE) digital diagnostic tool, https://pacev2.apexcovantage.com/. PACE helps ensure that figures meet PLOS requirements. To use PACE, you must first register as a user. Registration is free. Then, login and navigate to the UPLOAD tab, where you will find detailed instructions on how to use the tool. If you encounter any issues or have any questions when using PACE, please email PLOS at figures@plos.org. Please note that Supporting Information files do not need this step.
